# Does neck flexion improve performance? Effects on reaction time depend on whether responses are expected

**DOI:** 10.1007/s42452-023-05335-6

**Published:** 2023-03-17

**Authors:** Jason L. Baer, Rajal G. Cohen

**Affiliations:** 1 Department of Psychology and Communication Studies, University of Idaho, Moscow, ID, USA.

**Keywords:** Inhibition (psychology), Reaction time, Forward head posture, Ergonomics, Cognitive psychology, Embodied cognition

## Abstract

This research investigates the limitations of the apparent paradox in which neck flexion, which is associated with poor inhibition and neck pain, seems to facilitate performance in some tasks. We compared the effect of a flexed neck on performance in a reaction time and go-nogo task using a novel method of fixing neck posture. We hypothesize that using a flexed neck posture speeds response time for tasks with high prepotency (when participants are biased toward responding), but not for tasks with low prepotency (when participants are more likely to withhold a response). Previous findings demonstrated the effect of neck flexion on reaction time with a harness. In this study, participants complete both simple reaction time and go-nogo tasks with neck angles fixed in neutral or forward positions with tape. We found that simple reaction times were 10 ms faster in the forward neck position than in neutral; this facilitation was not seen in the go-nogo task. We conclude that using tape to induce a flexed neck posture facilitates reaction time during tasks that always require a response and does not affect reaction time on a task which may require withholding a response.

## 1 Introduction

Extensive research links a chronic flexed neck to chronic neck pain [1–3], especially during computer work [4, 5]. Efforts to improve sitting posture emphasize maintaining a neutral spine [6], but little attention is paid to underlying reasons people might deviate from these recommendations. One possible explanation originates in sports research on ready postures, showing that increased neck flexion can improve reaction time on motor control tasks [7].

Fujiwara and colleagues demonstrated that forward neck positions can reduce the threshold for sensory processing and muscle activation [8], leading to improved reaction times on saccade [9], antisaccade [10], and choice reaction time tasks [11]. In the aforementioned series of experiments, subjects put their heads forward prior to completing a set of reaction time tasks. The authors suggest that increases in muscle tone similar to those that occur during readiness for racing events can improve response time by reducing relevant sensory and motor thresholds. In these tasks, posture is fixed in place using a harness, making it difficult to identify how these effects may generalize to other tasks, particularly in non-laboratory conditions. Furthermore, all the previously studied tasks had high levels of prepotency, which may not reflect the nature of most real-life tasks.

Other studies where posture is not fixed in place suggest that a flexed neck posture does not always improve performance. Chronic forward head posture has been linked to reduced stability, worse balance on specific tasks, and decreased cervical proprioception [12]. The facilitation effect of neck flexion is not present in children under 11 years of age [13], and it is strengthened by training in a flexed neck position [9], suggesting that a learned association drives the effect of neck flexion on reaction time. In addition, we have observed that young adults whose habitual standing posture includes more neck flexion commit more false alarm errors during a go-nogo task than young adults with less neck flexion [14]. The apparent contradiction between our study associating flexed neck with worse performance and the studies associating flexed neck with better performance could be explained in one of two ways. First, Fujiwara and Kunita manipulated neck flexion, while our previous study was correlational. Maybe poor inhibitory control caused forward neck flexion in our participants, rather than the influence being in the other direction. Second, there may be key differences between the inhibitory control tasks used in each study.

Reaction time and inhibitory control tasks differ in how likely participants are to respond to presented stimuli, also referred to as prepotency [15, 16]. High prepotency reflects a bias toward responding to stimuli, and low prepotency reflects a bias toward withholding a response due to reduced certainty of the stimuli. Fujiwara and colleagues focused on response speed on tasks with high prepotency by measuring motor evoked potentials [8], saccadic reaction time [9], antisaccade reaction time [10, 17, 18], and choice reaction time [11]. For these tasks, the motor plan is likely to be already prepared because subjects are biased toward generating a response while anticipating stimuli [19, 20], and rapid *interruption* and cancellation is required for successful stopping [15]. In contrast, for inhibitory tasks with low response prepotency, *withholding* a response is more likely. For a go-nogo task, prepotency is characterized by the overall ratio of go and nogo cues presented to the participant, as well as the consistency of the interstimulus interval (ISI) [15]; for example, with 20% or more nogo trials the prepotency is significantly lower than in a simple reaction time task [21]. For a go-nogo task with this nogo rate, evidence suggests that a motor plan is NOT prepared before stimulus presentation, because the bias toward a response is lowered by the potential for false alarms; therefore, on successful nogo trials, the action is withheld [16].

By manipulating neck flexion during two timed computerized tasks with different levels of prepotency, we investigated the influence of posture on participants’ bias toward responding to stimuli. Because the previously observed facilitation effect of neck flexion involves readiness of a prepared action, we hypothesized the facilitation effect would be present for the simple reaction time (SRT) task and absent for a go-nogo task with 20% nogo trials. We predicted a faster SRT with a flexed neck than with a neutral neck, but no effect of neck flexion on go-nogo performance, assessed either by reaction time on hits or by percentage of false alarms.

The following section describes the experimental protocol for manipulating neck flexion (2.3), the measure for neck flexion (2.4.1), and the tasks completed by participants (2.4.2 and 2.4.3). In Sect. 3 we confirm the effectiveness of our experimental manipulation on posture (3.1) and assess its influence on each task (3.2). In Sect. 4 we present a summary of our findings (4.1), the theoretical implications (4.2), and practical implications (4.3) of the study. The discussion ends by presenting strengths of the study, limitations, and possible directions for future studies (4.4), followed by our conclusions (4.5).

## 2 Method

### 2.1 Equipment

**Fig. 1:**
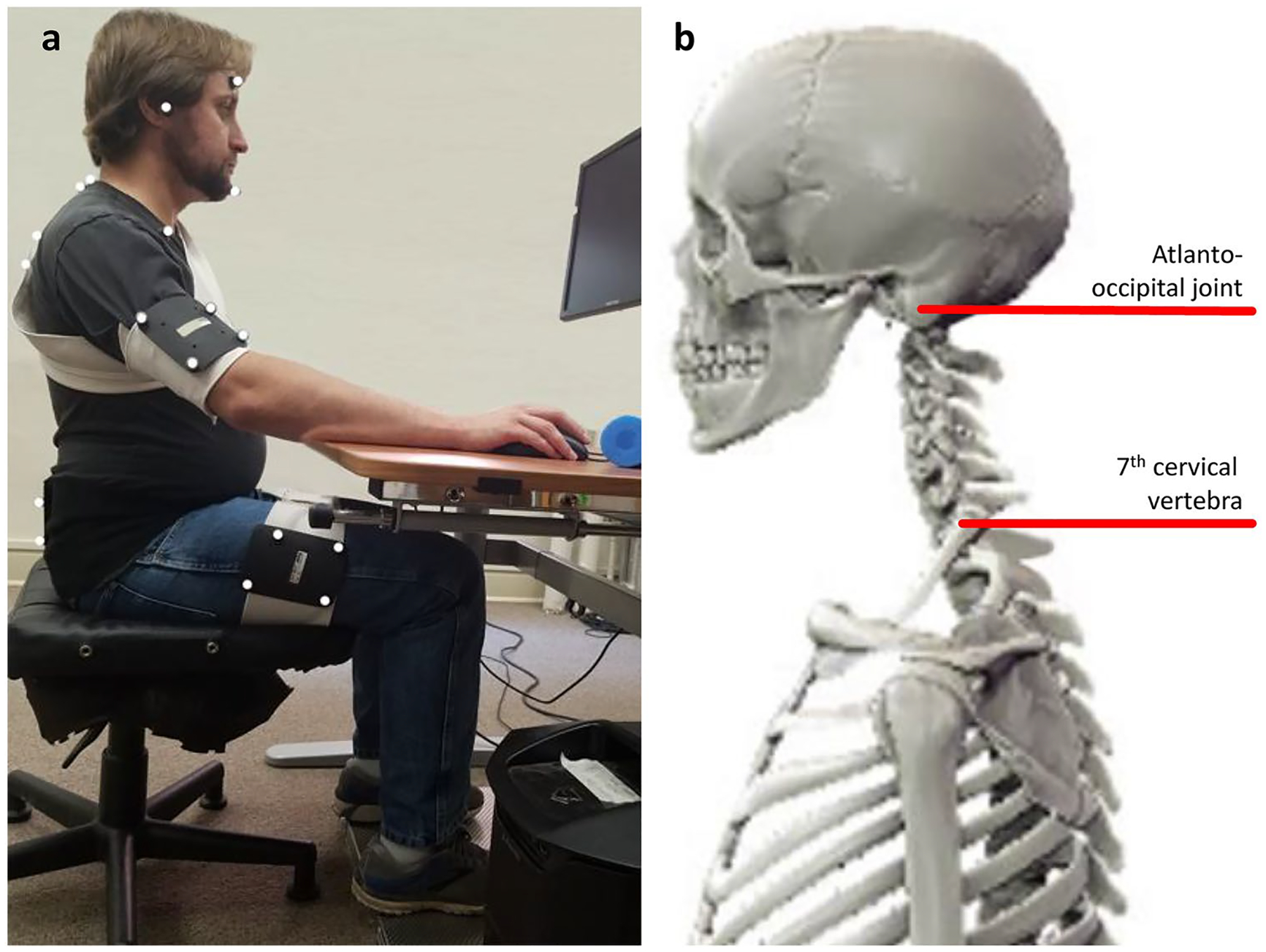
Setup: **a** First author sitting at adjustable workstation while wearing reflective marker clusters on the head, neck, upper torso, lower torso, left and right upper arm, and left and right thigh. **b** Composite model showing offset positions from surface landmarks for the atlanto-occipital joint and C7/T1 vertebra

To collect three-dimensional motion capture data, we placed 8 reflective marker clusters on participants’ body segments: head, neck (atlanto-occipital [AO] to C7/T1 joint), upper torso (C7/T1 to T12/L1 joint), lower torso (T12/L1 to L5/S1 joint), left and right upper arm, and left and right thigh (Fig. 1a). These segments were tracked with eight infrared Vicon Bonita motion capture cameras (Oxford, UK) at a rate of 100 frames per second. We used *The MotionMonitor* xGen software by Innovative Sports Training (Chicago, IL) to produce a composite model of each participant’s skeletal structure and joint centers based on offset positions from surface landmarks recorded in anatomical position [22]. The AO (between the left and right mastoid process), C7/T1 (7.18 cm forward, 3.35 cm down from the C7 spinous process), and L5/S1 (9.49 cm forward from the L5 spinous process) joint centers were used in analysis (Fig. 1b).

The participant workspace was equipped with an adjustable sit-stand desk (Rebel Crank-Up 1000), adjustable monitor display, and adjustable backless office chair. This allowed us to tailor the workstation to each participant’s anthropometric measurements. In addition, participants sat with their forehead against a chin rest that was modified to act as a forehead bar to control for seated distance from the screen in both neck position conditions and to prevent posture deviation. This forehead bar consisted of a padded stop for the chest, so that participants were unable to lean forward at the torso, and a Good-Lite table model chin rest, with the chin cup removed to provide a target position for the head during the task without interfering with neck flexion. The position of the metallic frame was adjusted for each participant to accommodate neutral and forward conditions.

To increase the ecological validity of the study, Transpore tape was placed across the neck to produce neck flexion angles similar to those seen in [7] without the use of a harness. Neck flexion angles were measured during pilot testing; the tape begins to pull on the skin if flexion deviates by less than one degree.

### 2.2 Participants

We tested 29 participants (13 men and 16 women) aged 18–24, recruited from psychology courses at the University of Idaho. This study was approved by the University of Idaho Institutional Review Board; all participants provided written informed consent and received course credit. Prior to testing, we screened participants for physiological and psychological issues that could interfere with their ability to perform the tasks. Participants were excluded from the study if they reported current musculoskeletal injuries (pain in any part of the body while standing or walking), neurological issues (diagnosed severe mental disorder), or any condition that could interfere with their ability to perform the task comfortably.

### 2.3 Protocol

After obtaining informed consent, experimenters adjusted the workspace for each participant using standard ergonomic guidelines [22] and attached reflective markers. Experimenters briefed participants on the importance of good sitting posture, indicating that ankles, knees, hips and elbows should rest at 90 degrees and the center of the screen should be less than 10 degrees below eye-level. Participants were instructed to maintain a neutral head position by allowing their heads to float at the top of their spines. We collected 10 s of baseline postural alignment data for the neutral posture according to these instructions. To collect baseline data for the forward posture, we asked participants to push their heads forward relative to their torsos by jutting their chins forward while looking at the computer screen.

**Fig. 2:**
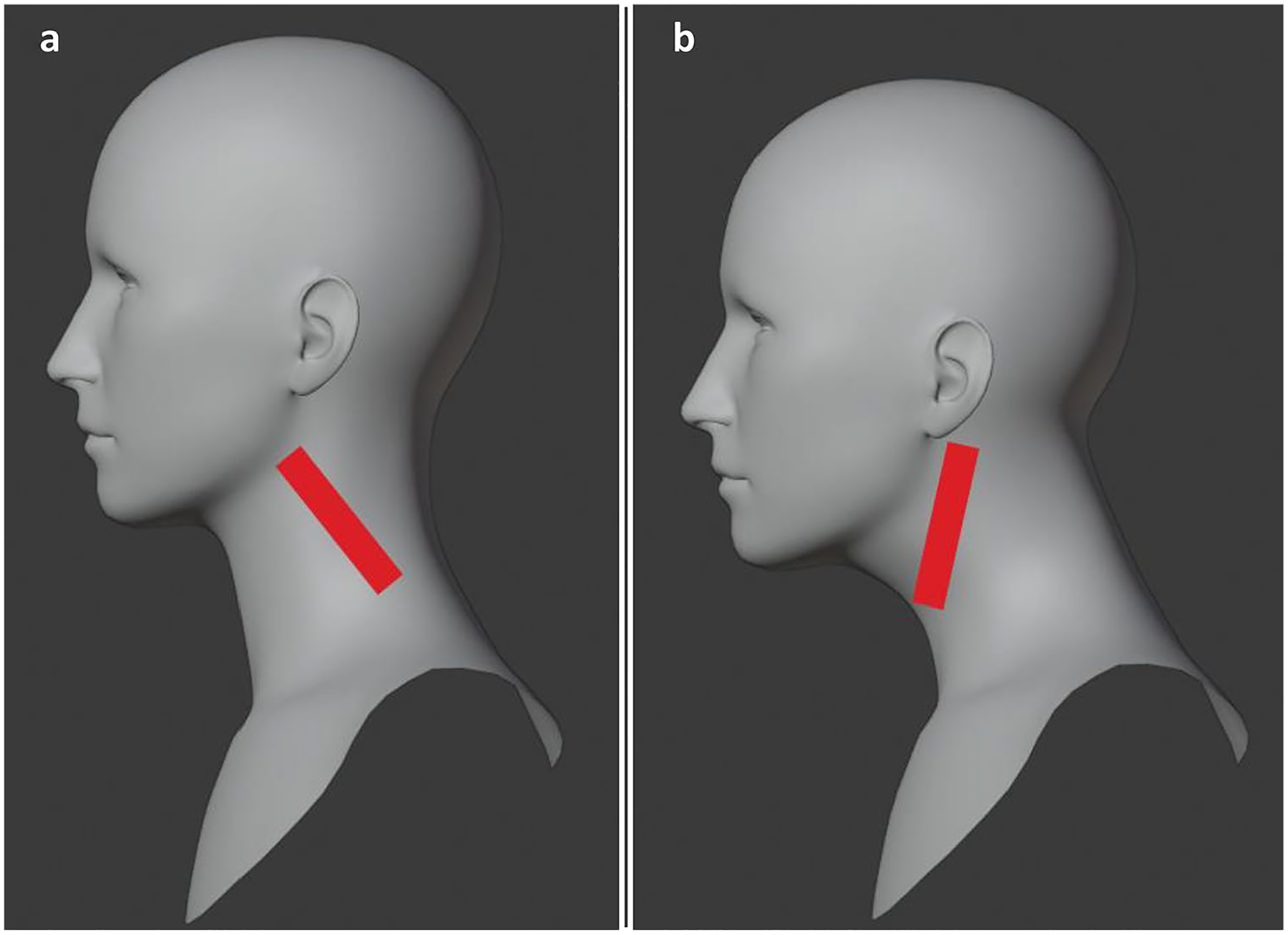
Tape position used to fix posture for **a** neutral and **b** forward conditions. **a** The tape pulls at the skin to prevent participants leaning forward at the head. **b** The tape pulls at the skin to prevent participants from straightening or moving the head back

To assure that participants maintained these postures while performing computer tasks, experimenters applied clear Transpore tape to the participants’ necks during each experimental condition. The tape was placed across surface muscles which would contract, causing the tape to pull at the skin when the participant began to shift their head forward (in the neutral neck position condition, Fig. 2a) or backward (in the forward neck position condition, Fig. 2b). Each participant practiced all computer tasks once without postural instruction. Before participants completed each task for either neck position, participants were reminded of the postural instructions and tape was applied. Previous studies of neck flexion demonstrated that the facilitation effect was present with at least 5 degrees of flexion compared to neutral [7]; to ensure the manipulation was consistent for our study, participants with a flexion increase of 3 degrees or less were excluded from analysis.

### 2.4 Measures

#### 2.4.1 Postural measures

**Fig. 3:**
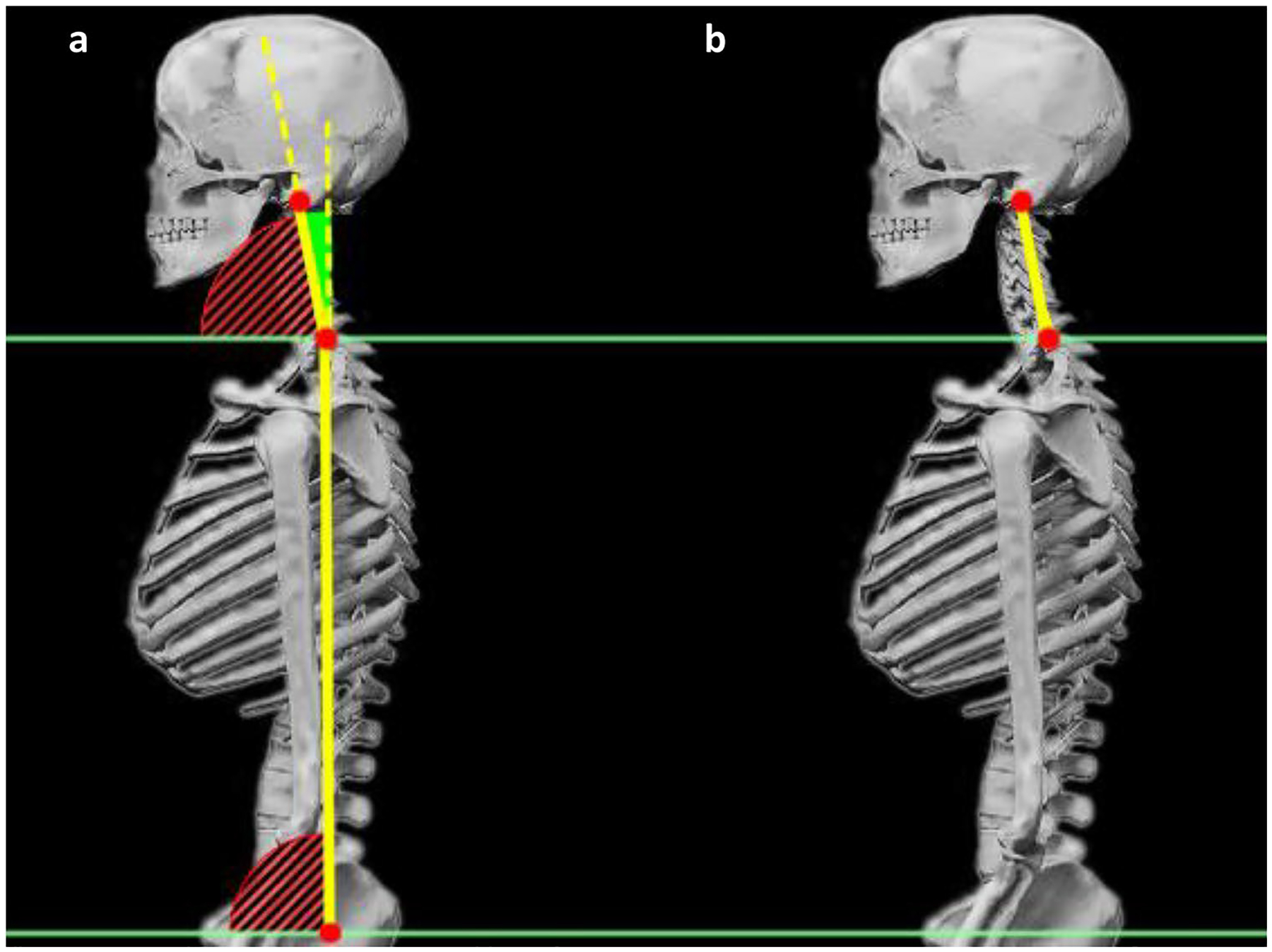
The Motion Monitor composite skeleton. **a** Digitized points for atlanto-occipital (AO) joint, C7/T1 vertebral joint, and L5/S1 vertebral joint are shown in red. Dependent measure of neck flexion angle is shown in solid green, and **b** neck length (distance between AO and C7/T1) is shown in yellow

We measured sagittal plane neck and torso angles in order to calculate flexion of the neck relative to the torso. See Fig. 3. The neck angle used a line from the midpoint of the mastoid processes to the C7 joint and then forward. The torso angle used a line from C7 joint to the joint of L5 and the first sacral vertebra (S1) and then forward. In all cases, a larger angle indicates greater extension. We defined neck flexion by subtracting the neck angle from the torso angle (Fig. 3a), giving a value that indicates neck flexion relative to the torso, where a more positive angle indicates a more forward head position relative to the torso. Due to anatomical variation, there is no universal neutral angle for the neck.

Additionally, we measured neck length to represent cervical spinal shrinkage, using the three-dimensional linear distance between the AO joint center and the C7/T1 joint center (Fig. 3b). Spinal shrinkage has been associated with pain and discomfort [23, 24]. In a previous study, greater neck disability correlated with increased neck shortening during a task [25].

#### 2.4.2 Simple reaction time task

For our task with high prepotency, all participants completed the simple reaction time task (SRT). In this task, participants were presented with a black computer screen, and once every 1.5–3.5 s (ISI varied randomly per trial in 500 ms intervals) they were presented with a single white capital letter in 36 point sans serif font. Participants responded to the stimuli by pressing the space bar with their dominant hand as fast as possible for 100 trials. Each letter remained visible for 250 ms; if participants failed to respond within 500 ms, the trial was counted as a miss. Reaction time was measured from the moment of stimulus presentation to the start of the button press, averaged across all successful trials. All participants completed each task twice (once in each neck position condition). The orders of tasks and conditions were counterbalanced using a Latin square.

#### 2.4.3 Go-nogo task

For our task with lower prepotency we used a go-nogo paradigm, where participants were presented with stimuli identical to those used in the SRT, with an ISI range of 1.5–3.5 s (500 ms intervals). Participants responded by pressing the space bar as quickly as possible, unless the letter presented was “X,” (the nogo stimulus) in which case they were supposed to withhold their response. There were 100 trials; 20% of these were nogo trials. Responses later than 500 ms after stimulus presentation were considered misses; false alarms were counted if the participant failed to inhibit their response to nogo stimuli. This task could be used to measure inhibition in three ways: average reaction time for hits, change in reaction time compared to SRT, and percentage of false alarms.

### 2.5 Statistical analysis

Data were processed using custom code written in MAT-LAB R2017a (Natick, MA); ANOVAs and correlations were conducted using SPSS Version 22. To demonstrate that the forward neck position produced greater neck flexion than the neutral neck position, we conducted a paired samples t-test for neutral and forward baseline postures. Levene’s test showed that homogeneity of variance was present for all dependent measures. Shapiro–Wilk tests showed that residuals were normally distributed for all dependent measures. Therefore, t-tests were justified.

To confirm that the postural difference was present during the tasks, we conducted a 2 × 3 ANOVA with dependent variable of neck flexion angle and factors of neck position (neutral and forward) and task (baseline, SRT, go-nogo). We also repeated the ANOVA with neck length as the dependent variable. Post-hoc comparisons were performed to assess pairwise differences between each task if significant main effects were found.

To test the prediction that increased neck flexion differentially improves reaction time on button press tasks, we conducted two-tailed paired-sample t-tests for SRT and go-nogo (reaction time and false alarms) in neutral and forward neck positions.

To test for the presence of the previously observed relation between inhibitory control and neck flexion, each measure of inhibitory control was correlated with participant’s baseline neck flexion angle using Pearson’s r.

## 3 Results

Data from four participants was excluded because they did not seem to understand experiment instructions (one participant had more than 70% false alarms on go-nogo; three participants had less than 3 degrees difference between the neutral and flexed neck positions).

### 3.1 Kinematics

**Fig. 4:**
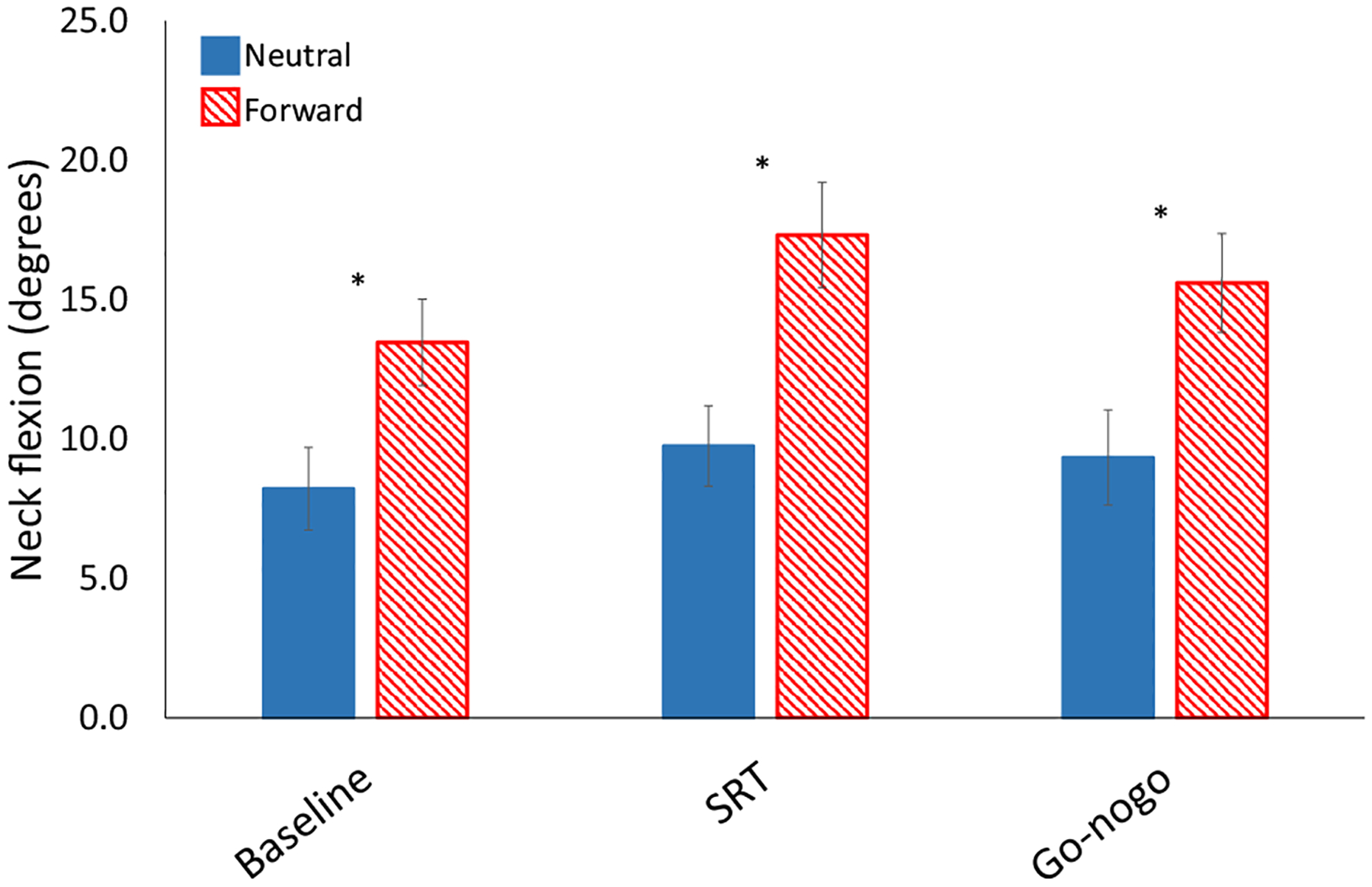
2 × 3 ANOVA comparing the effects of head position on neck flexion angle (degrees), with factors for condition (solid blue = neutral posture; red stripes = forward posture) and task (baseline, simple reaction time (SRT), and go-nogo). Error bars represent standard error. Asterisks represent significant effects (*p* < 0.05).* Main effect of condition showing that neck flexion was greater in the forward condition than in the neutral condition

The results of our experimental conditions on neck flexion are shown in Fig. 4. The use of tape to maintain posture led to a 5 degree increase in flexion during baseline for the forward position compared to neutral; *t*(24) = 6.9, *p* < 0.0001. During the computer tasks, this difference was maintained; *F*(1,24) = 12.4, *p* < 0.01, indicating that our manipulation was successful. There were no significant interactions.

**Fig. 5:**
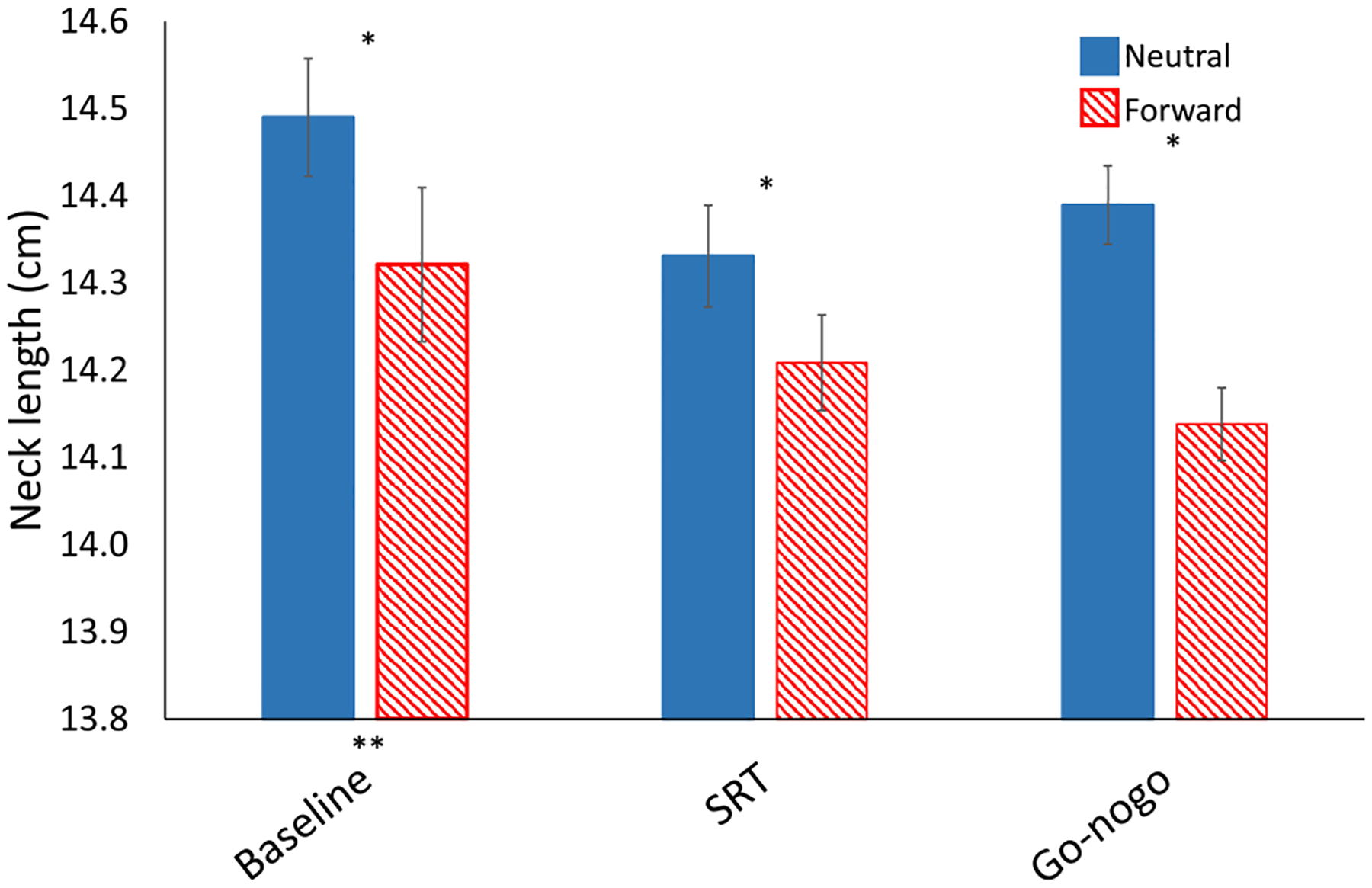
2 × 3 ANOVA comparing the effects of head position on neck length (cm), with factors for condition (solid blue = neutral; red stripes = forward) and task (baseline, simple reaction time (SRT), and go-nogo). Error bars represent standard error. Asterisks represent significant effects (*p* < 0.05). * Main effect of condition showing that participants’ neck length is shorter in the forward condition. ** Main effect of task on neck length showing participants neck length was shorter during SRT and go-nogo than baseline

The results of our experimental conditions on neck length are shown in Fig. 5. Overall, the use of tape to maintain posture led to a 2 mm decrease in neck length for the forward position compared to neutral; *F*(1,24) = 10.4, *p* < 0.01. There was also a main effect of task on neck length; *F*(2,48) = 6.7, *p* < 0.01. Post-hoc tests of simple effects showed that during tasks, participants’ neck length was one-quarter centimeter shorter than baseline on average for SRT, p = 0.02; and go-nogo, p = 0.01. There were no significant interactions.

### 3.2 Reaction time and inhibitory control tasks

**Fig. 6:**
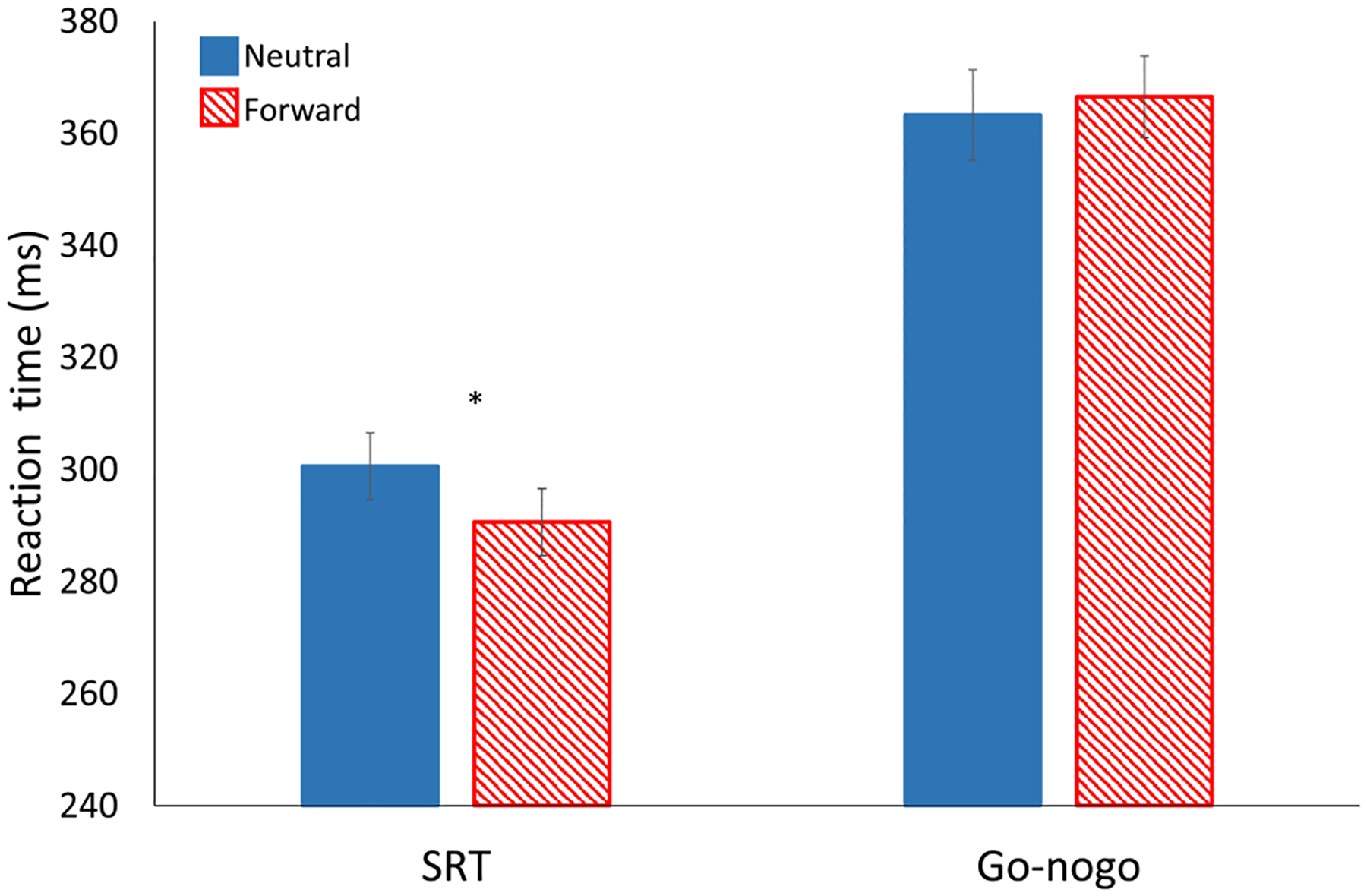
Reaction time during simple reaction time (SRT) and go-nogo tasks, for neutral (solid blue) and forward (red stripes) conditions. Reaction times are measured in ms from stimulus onset to button press. Error bars represent standard error. Asterisks represent significant effects (*p* < 0.05). Paired t-tests showed that reaction time was faster in the flexed neck condition for SRT, but there was no difference in reaction time for during go-nogo

Button press task performance scores are summarized in Table 1. The results of our experimental conditions on reaction time are shown in Fig. 6. Simple reaction times were 10 ms faster in the forward position than neutral; *t*(24) = 2.16, *p* = 0.04. In contrast, go-nogo RT and errors were not significantly affected by head position (3 ms slower in the forward position than neutral, with 2.9% fewer false alarm errors).

### 3.3 Relation between inhibitory control and posture

We detected a near-significant correlation between baseline neck flexion and false alarms during the go-nogo task (*r* = 0.40, two-tailed *p* = 0.05).

## 4 Discussion

### 4.1 Summary and interpretation

The goal of this study was to compare the effects of flexed neck posture and neutral posture on reaction time and inhibitory control. Previous research has shown that a flexed neck posture improves reaction time during anticipation and motor execution [8, 9, 11]; however, a flexed neck is also associated with poor inhibitory control performance [14]. This apparent disconnect led us to hypothesize that a flexed neck posture primes a go response which improves the speed of responses on tasks with high prepotency but would not improve reaction time during an inhibitory control task with low prepotency.

With respect to our initial predictions, we showed that a flexed neck improved reaction time but did not affect the go-nogo task. The faster reaction time seen here in the flexed neck condition compared to neutral posture is consistent with previous results, which showed that a flexed neck posture improves simple reaction time and antisaccade reaction time.

As predicted, we did not observe a facilitation effect of neck flexion on response time or accuracy for our go-nogo task. This is consistent with our hypothesis that a task with low prepotency would not be facilitated by a flexed neck posture. A go-nogo task with 20% nogo trials is different from simple reaction time, saccade, and antisaccade tasks because it allows participants to proactively decide not to move rather than changing an action in progress [15, 16]. Further, while Fujiwara and colleagues found that flexed neck posture facilitated performance in a task similar to ours [11, 15], they used a fixed ISI of 2.5 s, which likely increased prepotency relative to our variable ISI. The results of the present study are in line with the idea that when the motor plan to stop an initiated movement is not prepared before the stimulus is presented (as in a go-nogo task), neck flexion does not facilitate a response.

In comparison to our previous study, the correlation between forward head posture and false alarms on the go-nogo task was slightly weaker. This may be related to the previously established lower variability in sitting postures relative to standing postures [26].

### 4.2 Theoretical implications

Preparatory muscle contractions occur in advance of movement; this muscle contraction is thought to prime the execution of a motor plan [7], increase excitability of voluntary muscles and readiness to respond [11], increase focus within the central nervous system for detecting relevant stimuli [27], and increase brain activity in premotor cortex and supplementary motor area (SMA) [28]. This effect is likely diminished when the stimuli are difficult to anticipate [11, 28], as was the case in the go-nogo task used in the present study.

Reaction time and Go-nogo tasks recruit overlapping neural circuits within the right inferior frontal cortex (IFC), pre-SMA, and SMA. Evidence suggests that the decision to stop occurs in the IFG, and the SMA modulates motor activity. Prepotency may affect brain activation across areas of pre-SMA. In previous studies, reduced pre-SMA activity has been seen in go-nogo tasks compared to SRT tasks [15]. Neck flexion may provide feedback to these areas which influence reaction time for SRT but not go-nogo.

This research also intersects with the field of embodied cognition, where evidence suggests that changes in posture can affect cognition [29, 30]. Studies of mirror neurons show differences in cognition due to neurodegenerative disorders [29], and studies of expansive and contractive postures have elicited differing behavioral, emotional, and hormonal responses in participants [30]. However, previous studies of the influence of posture have looked at more “high level” aspects of cognition and behavior, such as risk taking, rather than low-level aspects such as response inhibition. The forward neck posture used in this study and in the studies by Fujiwara and colleagues bears some resemblance to the contractive postures used in studies of embodied cognition. Therefore, it is possible that the effect seen here, in which forward neck flexion enhanced prepotent tendencies may help explain previous findings that contractive postures are weakly associated with increased cortisol [30], and may be associated with increased sympathetic nervous system activation.

### 4.3 Practical implications

Our results provide insight into possible reasons people may adopt a flexed neck posture when under pressure at work. Extensive research links chronic flexed neck posture (often termed forward head posture) in computer work with chronic neck pain [5, 31–33]. Sitting with a flexed neck posture leads to increased compression of the vertebrae and increases the mechanical load on the spine. This extended stress on the neck compresses nerves, leading to pain and strain on surrounding muscle tissue. All this leads to the question: why do people sit this way, if it is so bad for them? One possible answer is that the short-term benefits of faster response times may be more salient than the long-term costs to health.

People adapt their posture in response to the demands of tasks [14, 16, 34]. For example, the demand to move quickly in an athletic context may evoke muscle tension that induces neck flexion [7]. This tension/flexion pattern may also provide speed benefits in the context of a computer task as seen in this study. Previous studies of this effect used a complex harness. In order to study the phenomenon in a more ecologically valid way, we used a simpler method for fixing posture. The present study demonstrates that the effect of neck flexion can be observed without the use of a complex harness, opening the door for future studies of the effects of acute neck flexion. It would be beneficial to determine if the facilitation effect of moderately flexed neck posture on reaction time seen in this study would generalize to tasks common during office work, or whether everyday computer tasks tend to be more similar to the go-nogo task used here, where performance did not benefit from neck flexion.

Understanding potential benefits of a flexed neck posture despite its association with long term neck pain may improve our understanding of why poor posture develops, thus providing crucial foundational knowledge for those seeking to develop effective postural correction to remedy neck pain. In particular, effective posture correction may depend on the client’s willingness to let go of performing as fast as possible, and this may need to be made explicit. Although the absolute difference in reaction time between the two neck positions in this study was small (around 10 ms), the percent difference was substantial and would probably be perceptible to an office worker under pressure to complete work quickly. Of course, most real world tasks are not as simple as a pure reaction time task, so this facilitation effect might not be as strong. Further evidence that people are willing to sacrifice a neutral head posture in the interest of completing a task can be seen in our previous work showing that neck flexion increases in anticipation of target-directed stepping [14].

### 4.4 Strengths, limitations, and future directions

Our experimental design was based on the methodology laid out by Fujiwara and colleagues [8, 9, 11], modified to provide greater ecological validity. Although our use of tape to affect neck posture did not allow the same degree of control as the harness used in other studies, our manipulation check demonstrated that it led to a 5° difference between neutral and forward postures. While a difference of 5 degrees seems small, it is similar to that seen in previous research by Fujiwara and colleagues [7]. The presence of tape on the neck may have introduced a distraction which could have affected results. However, the tape was present in both neck positions, and it is likely that any way to fix posture would produce similar attentional demands.

In this study, we used fixed task parameters for two separate tasks to allow us to test the influence of high and low prepotency on reaction time. However, prepotency cannot be measured directly, so it is not possible to quantify how prepotent responses were in the two tasks. In other lines of research, increased nogo stimulus probability has correlated positively with brain activity related to decision making processes [21]. Therefore, we might expect that a flexed neck posture would have a greater effect on go-nogo performance in a task with a lower rate of nogo stimuli than was used in the present study. A future study could vary the nogo stimulus rate or the predictability of the ISI in a go-nogo task to further explore how manipulating the prepotency of a task affects the facilitation of response times by a flexed neck posture.

Preparatory muscle activity in response to stimuli provides a clearer picture of reaction time than button press activity [35]; thus a future study could add EMG. Similarly, the addition of EEG could allow us to ascertain whether the facilitation effect of neck flexion on SRT but not go-nogo would be associated with increased SMA activity in SRT but not go-nogo.

### 4.5 Conclusion

This study assessed the influence of neck posture on reaction time in tasks with different levels of prepotency, comparing a neutral posture with a flexed neck posture. Flexed neck posture facilitated response time during a task with high prepotency but did not facilitate response time or accuracy on a less prepotent task that allowed for proactive inhibitory control. This supports our hypothesis that neck flexion only facilitates responses when the motor plan is prepared before the stimuli are presented and suggests limitations on the benefits of this posture in real-life scenarios.

## Figures and Tables

**Table 1:** Effect of head position on reaction times and errors

Task Measure	SRT Reaction time (ms)	Go-nogo	
		GnG RT (ms)	GnG FA (%)
Neutral	300.6 ± 29.9	363.3 ± 39.7	36.9 ± 16.5%
Forward	290.7 ± 29.9	366.6 ± 35.8	34.0 ± 13.8%
*t* (df)	2.16 (24)	1.09 (24)	0.51 (24)
*p*-value	0.04*	0.61	0.28

Effects of head position condition (rows) on responses to reaction time and inhibitory control measures (columns) for button press tasks: mean ± standard deviation. Asterisks denote significant effects of neck position (*p* < 0.05). Measures: Simple reaction time (SRT), go-nogo reaction time (GnG RT), and go-nogo false alarm responses (GnG FA)

## Data Availability

The datasets generated during and/or analyzed during the current study are available from the corresponding author on reasonable request.

## Article highlights

- We investigated the influence of neck flexion on two time-sensitive tasks with differing prepotency.
- A flexed neck led to faster RT on a high prepotency task but not on a task with low prepotency.
- This study replicates previously seen effects of neck flexion on high prepotency RT, with higher ecological validity.

## Abbreviations

AO: Atlanto-occipital joint
C7: Seventh Cervical Vertebra
GnG FA: Go-nogo false alarms (percentage)
GnG RT: Go-nogo reaction time (average in milliseconds)
T1: First thoracic vertebra
T12: Twelfth thoracic vertebra
L1: First lumbar vertebra
L5: Fifth lumbar vertebra
S1: First sacral vertebra (sciatica)
SRT: Simple reaction time (average in milliseconds)
RTΔ: Reaction time change (GnG RT—SRT)
